# Machine‐learning prediction of urine‐culture positivity in a multicentre test‐ordered cohort: Model development and internal validation

**DOI:** 10.1002/bco2.70280

**Published:** 2026-09-08

**Authors:** Isaac Samir Wasfy, Jamal Ahmad, Hany Elsegeay, Mohamed F. Elebiary, Ahmed Haty, Eman M. El‐Dydamony, Ahmed Mohamed Soliman, Ahmed Alrefaey, Hesham Abozied, Mohamed Algammal, Hossam A. Shouman, Maha M. Elzamek, Ahmed Abdel Galil Saleh, Ahmed Fetyan Abdelazim Shafi, Osama Mostafa Abdalla Mohamed

**Affiliations:** ^1^ Urology Department, Faculty of Medicine Suez Canal University Ismailia Egypt; ^2^ College of Medicine and Health Sciences Palestine Polytechnic University Hebron Palestine; ^3^ Urology Department, Faculty of Medicine Al‐Azhar University Asyut Egypt; ^4^ Urology Department, Faculty of Medicine Al‐Azhar University Cairo Egypt; ^5^ Urology Department, Faculty of Medicine for Girls Al‐Azhar University Cairo Egypt; ^6^ Urology Department, Faculty of Medicine Fayoum University El‐Fayoum Egypt

**Keywords:** antibiotic stewardship, clinical decision support system, machine learning, natural language processing (NLP), predictive modelling, urinary tract infection (UTI), urine culture

## Abstract

**Objectives:**

This study aim to develop, compare and internally validate machine‐learning models for predicting urine‐culture positivity in patients who had both urinalysis and culture ordered and to explore descriptive probability strata. Post hoc secondary analyses examined age subgroups, the incremental contribution of text‐derived features, simpler comparators and calibration.

**Patients and Methods:**

Urine culture results are typically unavailable for 24–72 h, creating uncertainty during initial assessment, and machine‐learning models may help estimate the probability of culture positivity from routinely collected data. This retrospective study included 2530 urine‐sample records originating from three university hospitals. Eligibility was based on paired urinalysis and urine culture records rather than symptom‐based diagnostic criteria for urinary tract infection (UTI). Thirteen supervised algorithms were evaluated using a stratified 75:25 sample‐record split. This constituted internal validation; records were not grouped by patient or centre because stable cross‐centre patient, centre and collection‐date identifiers were unavailable.

**Results:**

Several gradient‐boosting algorithms showed similar discrimination. CatBoost had the numerically highest test‐set AUC of 0.858 (95% CI 0.829–0.892), but its AUC did not differ significantly from gradient boosting or XGBoost. At the reported operating threshold, sensitivity was 0.587 (95% CI 0.513–0.662), specificity 0.930 (0.903–0.951), PPV 0.766 (0.693–0.833) and NPV 0.851 (0.821–0.882). Exploratory probability strata separated records with different observed rates of culture positivity, but their clinical utility and safety were not evaluated.

**Conclusion:**

Machine‐learning models discriminated between culture‐positive and culture‐negative sample records in a sample‐level internal validation. Culture positivity is not synonymous with symptomatic or clinically significant UTI. The findings support further evaluation as diagnostic risk‐estimation tools after urinalysis results are available. They do not establish clinical UTI or the safety or effectiveness of starting, withholding or delaying antibiotics. Patient‐grouped external validation with clinical outcomes is required before clinical use.

## 1 INTRODUCTION

Urinary tract infections (UTIs) are one of the most common bacterial infections worldwide, affecting 150 million people annually and creating a significant economic burden.^1^ A persistent challenge in clinical practice is the accurate and timely diagnosis of UTIs. Clinicians are often caught between the variable reliability of rapid diagnostic tests, such as urinalysis for nitrites and leukocyte esterase, and the inherent 24–72‐h delay for definitive urine culture results.^2^ This uncertainty can contribute to empirical antibiotic prescribing when bacterial UTI is clinically suspected. Treatment decisions nevertheless depend on symptoms, illness severity, host factors and alternative diagnoses; neither a positive culture nor a model prediction alone establishes symptomatic infection. Studies report that a substantial proportion of prescriptions made under diagnostic uncertainty may be unnecessary, contributing to antimicrobial resistance.^3^ Contemporary guidelines recommend population‐ and syndrome‐specific use of urine culture rather than universal culture for urinary symptoms. In adults, culture is recommended for suspected systemic UTI, persistent or recurrent symptoms, atypical presentations, increased risk of resistant organisms and pregnancy; NICE also recommends culture before antibiotics in men and pregnant women with symptomatic lower UTI. In children, indications depend on age, illness severity and dipstick findings, including age under 3 months, suspected upper UTI, serious illness risk, positive leukocyte esterase or nitrite, recurrent or nonresponsive infection or discordance between symptoms and dipstick results. Routine culture and treatment of asymptomatic catheterized patients are discouraged, and asymptomatic‐bacteriuria screening is generally reserved for pregnancy and selected endoscopic urological procedures involving mucosal trauma.^4^, ^5^, ^6^, ^7^, ^8^, ^9^, ^10^

The advent of machine learning (ML) offers a powerful opportunity to overcome these diagnostic limitations.^11^ Unlike traditional statistical models that often assume linear relationships, ML algorithms can identify complex, non‐linear patterns within large clinical datasets.^12^ Recent advances in Natural Language Processing (NLP) further enhance this capability by enabling the extraction of valuable information from unstructured free‐text clinical notes, capturing nuanced symptoms and context that structured data alone cannot represent.^13^ While several studies have highlighted the potential of ML in UTI diagnosis, they have predominantly been conducted in Western healthcare systems, limiting their generalizability.^14^ A structured comparison of these studies is provided in Table S1, and selected machine‐learning applications in antimicrobial‐resistance prediction are summarised in Table S2.

This study used pooled records from three Al‐Azhar University teaching hospitals to evaluate machine‐learning prediction in a Middle Eastern test‐ordered cohort.^15^ The primary objective was to develop, compare and internally validate models for the supplied urine‐culture label. Secondary objectives were to describe exploratory probability bands and, in post hoc analyses, compare age subgroups, quantify the contribution of text‐derived features, assess simpler baselines and examine calibration.

Because the model uses urinalysis results, its intended prediction time is after urinalysis becomes available but before the final culture result in patients who have already undergone culture. It was not designed to decide whether culture should initially be ordered, diagnose symptomatic UTI or direct antibiotic treatment. No software interface or medical device was deployed; workflow, cost‐effectiveness, treatment effects and safety were not evaluated. Any future use would require prospective implementation and safety evaluation.^16^

## 2 METHODS

### 2.1 Study design and data source

This multicentre‐source retrospective cohort comprised 2530 urine‐sample records generated during 2024 at three Al‐Azhar University teaching hospitals and included in the source extracts because paired urinalysis and urine‐culture results were available. No symptom‐based eligibility criterion was applied; the source records included urinary complaints, non‐urinary complaints, antenatal testing, preoperative testing and routine check‐ups. The term “suspected UTI” therefore denotes a pragmatic test‐ordered population rather than a cohort with clinically adjudicated symptomatic UTI. The de‐identified pooled extract did not retain hospital identifier, collection date, inpatient/outpatient or emergency setting, clinician‐stated indication, catheter status, pregnancy as a structured variable or specimen‐collection method. Accordingly, “multicenter” describes provenance only; centre‐specific, leave‐one‐centre‐out, temporal and setting‐specific performance could not be assessed.

The study protocol was designed to adhere to the Transparent Reporting of a multivariable prediction model for Individual Prognosis or Diagnosis (TRIPOD) guidelines to ensure clarity and reproducibility.^17^ Given the retrospective and de‐identified nature of the data, the institutional review board waived the requirement for individual patient consent.

### 2.2 Study population and outcome

The analytic unit was one dataset row representing a urine‐sample record. The 2530 rows cannot be assumed to represent 2530 unique patients or encounters. A file number was present in 2529 records, comprising 2294 distinct values; 176 file numbers occurred more than once across 411 records. Because centre and collection‐date identifiers were unavailable, repeated values could represent repeat sampling, separate encounters or reuse of locally assigned identifiers at different sites. All rows were retained and the primary split was performed at the sample‐record level without patient grouping. Possible within‐person correlation and train–test overlap are therefore limitations.

The prediction target was the binary culture classification in the supplied laboratory extracts, not clinically adjudicated UTI. Records in the “+ve Growth” extract were coded positive and records in the “No Growth” extract were coded negative; no record was reclassified post hoc according to colony count, organism, symptoms, pyuria, sex, age, catheter status or specimen method. Among 715 positive‐labelled records, the CFU field was populated in 684 and missing in 31; 10 records contained a count below 10 000 CFU/mL, 40 contained a count above 100 000 CFU/mL, and the remaining 634 had recorded counts between 10 000 and 100 000 CFU/mL. Organism data were missing in 11 positive records; nine records contained Candida and six listed more than one organism. The negative extract contained no organism, CFU or explicit contamination field, so mixed‐flora and contamination handling could not be verified. No single colony‐count threshold defines UTI across all populations and specimen types, and the clinical variables needed to distinguish symptomatic UTI, asymptomatic bacteriuria, colonisation and contamination were unavailable. The outcome must therefore be interpreted strictly as the supplied culture label.^4^, ^6^, ^7^

### 2.3 Predictor variables and feature engineering

A total of 22 features were used as predictors for the machine learning models. These variables were extracted and engineered from the raw data and were grouped into three distinct types:

1. Numerical Features (*n* = 3): Patient age, white blood cell (WBC) count from urinalysis and red blood cell (RBC) count from urinalysis.
2. Categorical Features (*n* = 4): Patient gender and the dichotomous results from the urinalysis dipstick for blood, nitrite and leukocyte esterase.
3. Natural Language Processing (NLP) Features (*n* = 15): Text was constructed by concatenating the available C/P, past medical history and past surgical history fields. These fields should not be interpreted as a complete chief complaint and history of present illness.

The primary analysis reported standardisation of numerical variables, numerical encoding of categorical variables and TF‐IDF representation of text. A post hoc data audit identified mixed‐unit age entries and one malformed gender value. Corrected descriptive summaries used unit‐aware conversion of days, weeks and months to years. Raw predictor missingness was present for blood (*n* = 2), nitrite (*n* = 85), leukocyte esterase (*n* = 85), RBC (*n* = 85) and WBC (*n* = 86); the statement that no missing data were present was therefore removed. The fitted primary preprocessing objects were unavailable for independent audit. In the post hoc reconstruction reported below, numerical and categorical imputation, scaling and TF‐IDF fitting were estimated using the training data only and then applied unchanged to the test set. Text was lowercased, English stopwords were removed, and the 15 retained tokens were ‘abd’, ‘check’, ‘fever’, ‘git’, ‘htn’, ‘known’, ‘luts’, ‘non’, ‘origin’, ‘pain’, ‘routine’, ‘sp’, ‘storage’, ‘troubles’ and ‘upper’. No dedicated negation detection, spelling normalisation, abbreviation expansion or language‐identification procedure was used. The dataset contained no note timestamps, so the timing of text relative to sampling, urinalysis reporting, culture finalisation and antibiotic prescribing could not be verified; temporal leakage cannot be excluded.

### 2.4 Model development and validation

The dataset was divided into a training set (*n* = 1897; 75%) and a hold‐out test set (*n* = 633; 25%). Stratified sampling was used to ensure that the prevalence of positive urine cultures (28.3%) was preserved in both subsets. This corresponded to 536 positive and 1361 negative cultures in the training set and 179 positive and 454 negative cultures in the test set.

Thirteen distinct supervised machine‐learning algorithms were trained and evaluated to provide a comprehensive comparison across different model families:

- Linear model: Logistic egression
- Kernel‐based model: Support vector classifier (SVC)
- Probabilistic Model: Naive Bayes
- Instance‐based model: K‐nearest neighbours (KNN)
- Tree‐based ensembles (bagging): Decision tree, random forest, extra trees
- Tree‐based ensembles (boosting): AdaBoost, gradient boosting, XGBoost, LightGBM and CatBoost
- Neural network: A multilayer perceptron model

For the primary model comparison, hyperparameter optimization was reported as tenfold stratified cross‐validation within the training partition using RandomizedSearchCV with 20 parameter combinations, followed by evaluation in the sample‐record hold‐out partition. The saved search objects, fitted preprocessing objects and individual prediction vector were not retained in the supplied archive. The centres had already been pooled, and the split was not grouped by file number; possible dependence among repeated records may therefore have produced optimistic estimates. This design constitutes sample‐level internal validation only and does not provide geographic, centre‐level, patient‐level or temporal validation.

### 2.5 Performance evaluation and statistical analysis

The final performance of each tuned model was assessed on the sample‐record hold‐out test set. A comprehensive suite of metrics was calculated to characterize discrimination, classification performance and overall probabilistic accuracy:

- Discrimination metrics: Area under the receiver operating characteristic curve (AUC‐ROC), accuracy, F1‐score, sensitivity (recall), specificity, positive predictive value (PPV) and negative predictive value (NPV).
- Overall probabilistic accuracy: The Brier score summarised the mean squared error of predicted probabilities; lower values indicate better overall probabilistic accuracy, but the Brier score alone does not establish calibration.
- Likelihood‐ratio metrics: Positive likelihood ratio (LR+), negative likelihood ratio (LR−) and the diagnostic odds ratio (DOR) were calculated to describe how a model result changed the odds of the reference outcome—urine‐culture positivity—not the odds of clinically confirmed infection.

For the primary model comparison, 95% confidence intervals were estimated using 500 bootstrap resamples of the sample‐record test set and the DeLong test was used for pairwise AUC comparisons. The age‐subgroup, NLP‐ablation, simpler baseline and extended‐calibration analyses were conducted post hoc in response to peer review; they were not prespecified in the original protocol. These analyses used a separately reconstructed 75%/25% sample‐record split and CatBoost workflow with fixed hyperparameters, unit‐aware age parsing and preprocessing fitted on the reconstructed training partition. AUC confidence intervals used 2000 bootstrap resamples, and the paired NLP comparison used 3000 resamples. Because the reconstructed full model had AUC 0.869 rather than the primary model's 0.858, its calibration and ablation estimates are reported explicitly as post hoc reconstruction results and are not combined with the primary confusion matrix or probability‐stratum counts. Subgroup sensitivity and specificity were calculated at the training‐set Youden threshold (0.389).

### 2.6 Exploratory probability strata

For descriptive interpretation of the archived primary CatBoost output, predicted probabilities were divided into three exploratory bands: <0.20, 0.20–0.60 and >0.60. The provenance of these cut‐points could not be verified from the retained analysis materials; it is therefore unknown whether they were selected before or after inspection of model predictions or test‐set performance. They should be treated as post hoc descriptive categories, not as prespecified or clinically derived thresholds. They were not selected using decision‐curve or net‐benefit analysis and have not been clinically validated. No antibiotic or other clinical action is assigned to any band.

- Lower predicted probability: <0.20
- Intermediate predicted probability: 0.20–0.60
- Higher predicted probability: >0.60

The descriptive separation of these bands was summarised using the number of sample records and observed culture‐positive proportion in each category.

### 2.7 Ethics statement

The study protocol was reviewed and approved by the Institutional Review Board (IRB) of Al‐Azhar University (approval number: DFM‐IRB 00012367‐25‐07‐015). The IRB waived the requirement for individual patient consent, given that the study was a retrospective analysis of de‐identified data. All procedures were performed in compliance with the ethical standards of the institutional research committee and the 1964 Helsinki Declaration and its subsequent amendments.

## 3 RESULTS

### 3.1 Cohort characteristics

The analysis included 2530 urine‐sample records: 715 were classified culture‐positive (28.3%) and 1815 culture‐negative (71.7%). Median age was 11 years (IQR 5–37; range approximately 3 days–94 years). Age‐group distribution was 1325 (52.4%) aged <12 years, 242 (9.6%) aged 12–17 years, 834 (33.0%) aged 18–64 years and 129 (5.1%) aged ≥65 years (Table 1). Median age was 13 years (IQR 2.5–44.5) in the positive group and 10 years (IQR 5–34) in the negative group. The C/P field was populated in 2523 records, whereas past medical history was populated in only 290 (11.5%) and blank in 2240 (88.5%); a blank entry could not be interpreted as absence of comorbidity. One gender value was malformed and treated as unclassified for descriptive reporting. These characteristics emphasise the predominantly paediatric, clinically heterogeneous nature of the test‐ordered cohort. Care setting was unavailable, and the age structure should not be attributed specifically to an outpatient or urology‐service case mix.

**TABLE 1:** Baseline characteristics of the study cohort.

Characteristic	Category	Value
Total Samples		2530
Culture result	Positive	715 (28.3%)
Culture result	Negative	1815 (71.7%)
Recorded gender	Male	1168 (46.2%)
Recorded gender	Female	1361 (53.8%)
Age group (years)	<12	1325 (52.4%)
Age group (years)	12–17	242 (9.6%)
Age group (years)	18–64	834 (33.0%)
Age group (years)	≥65	129 (5.1%)
Top 5 presenting complaints (C/P)	Non‐specific abdominal pain	553
Top 5 presenting complaints (C/P)	Routine check‐up	552
Top 5 presenting complaints (C/P)	Fever of unknown origin	472
Top 5 presenting complaints (C/P)	Storage LUTS	211
Top 5 presenting complaints (C/P)	Antenatal care	78
Most frequent documented comorbidity mentions	Hypertension (HTN)	140
Most frequent documented comorbidity mentions	Diabetes mellitus (DM/NIDDM/IDDM)	89
Most frequent documented comorbidity mentions	Asthma	25
Most frequent documented comorbidity mentions	Sickle cell disease	14
Most frequent documented comorbidity mentions	HIV	10

*Note*: Values for culture result, recorded gender and age group are number (%) of the total cohort (*n* = 2530); one malformed gender value was classified as unreported, so displayed male and female counts sum to 2529. Age entries recorded in days, weeks and months were converted to years before grouping. Presenting‐complaint counts use case‐ and whitespace‐normalised whole‐field labels; composite complaint entries were retained as separate categories, and seven records had no C/P entry. Comorbidity counts are nonmutually exclusive keyword‐normalised mentions from the free‐text past‐medical‐history field, which was populated in 290 records (11.5%) and blank in 2240 (88.5%); they must not be interpreted as prevalence estimates.

Abbreviations: C/P, chief/presenting complaint; DM/NIDDM, diabetes mellitus/non‐insulin‐dependent diabetes mellitus; HIV, human immunodeficiency virus; HTN, hypertension; LUTS, lower urinary tract symptoms.

### 3.2 Comparative performance of machine learning models

**FIGURE 1:**
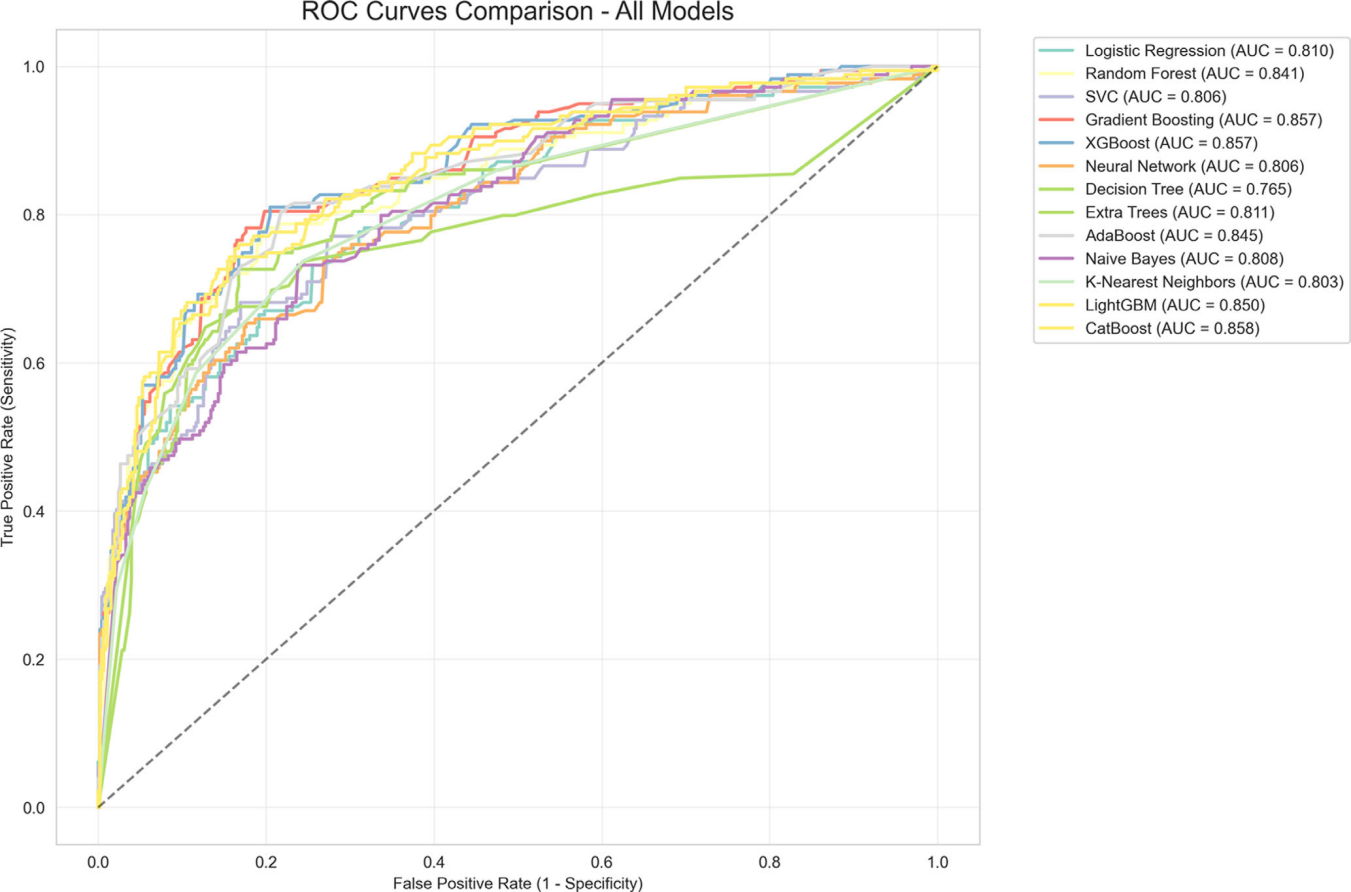
Receiver operating characteristic (ROC) curves for all models.

**FIGURE 2:**
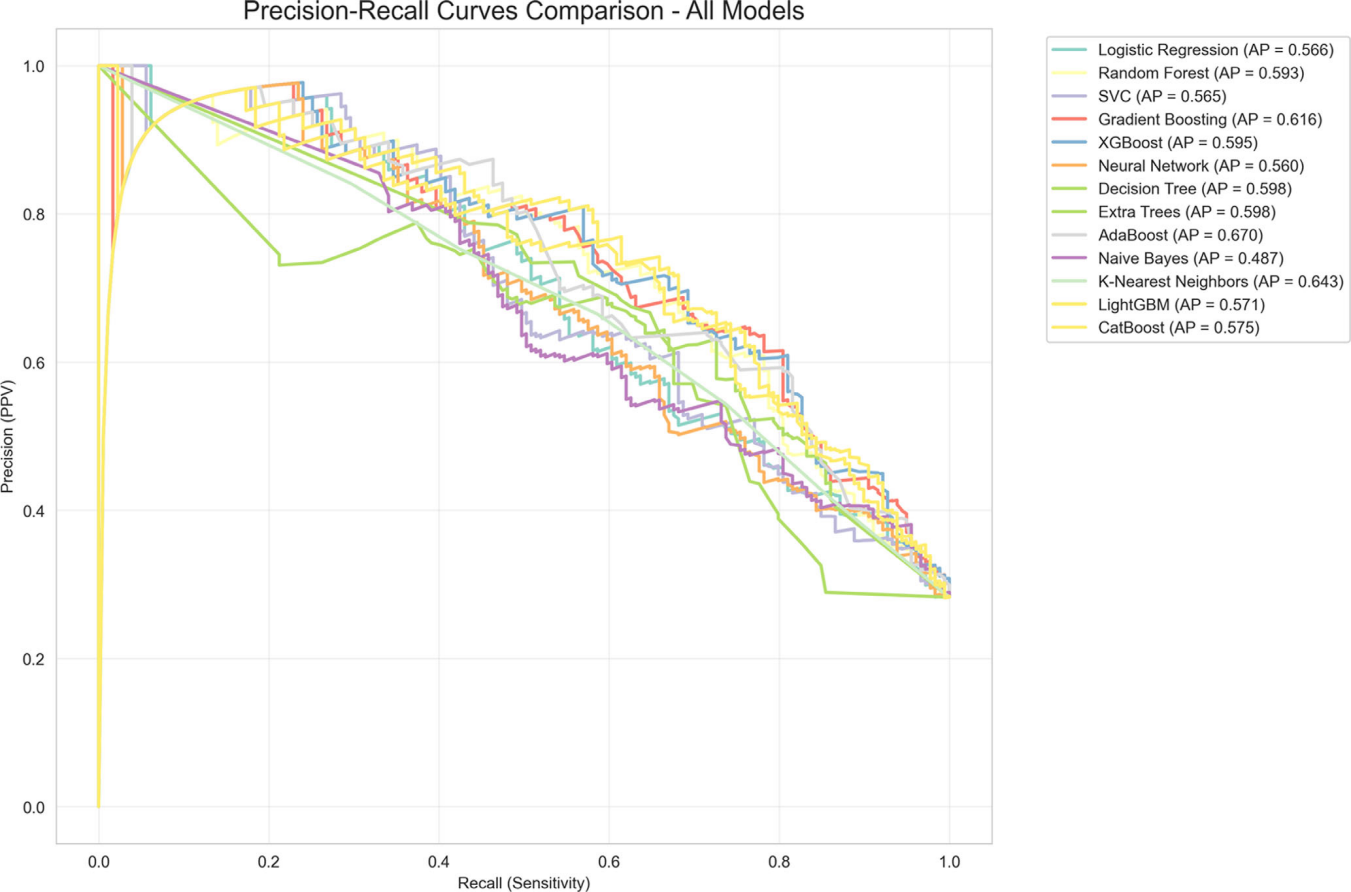
Precision–recall (PR) curves for all models.

All 13 machine‐learning models were trained and evaluated in the primary analysis. Gradient‐boosting algorithms had the highest numerical AUCs, although the estimates for the leading models were closely similar (Table 2; Figure 1). Precision–recall curves, which are more informative than receiver operating characteristic curves at the 28.3% outcome prevalence of this cohort, showed the same ordering of the leading models (Figure 2). A heat map summarising all performance metrics across the 13 models is provided in Figure S1.

**TABLE 2:** Performance metrics of all 13 machine learning models on the test set.

Model	AUC (95% CI)	Accuracy (95% CI)	Sensitivity (95% CI)	Specificity (95% CI)	PPV (95% CI)	NPV (95% CI)	Brier score (95% CI)
CatBoost	0.858 (0.829–0.892)	0.833 (0.806–0.861)	0.587 (0.513–0.662)	0.930 (0.903–0.951)	0.766 (0.693–0.833)	0.851 (0.821–0.882)	0.125 (0.107–0.142)
Gradient boosting	0.857 (0.823–0.888)	0.828 (0.802–0.858)	0.547 (0.477–0.620)	0.938 (0.917–0.959)	0.778 (0.711–0.847)	0.840 (0.812–0.870)	0.126 (0.111–0.143)
XGBoost	0.857 (0.826–0.891)	0.836 (0.804–0.866)	0.570 (0.497–0.647)	0.941 (0.916–0.960)	0.791 (0.716–0.857)	0.847 (0.812–0.878)	0.126 (0.109–0.144)
LightGBM	0.850 (0.814–0.883)	0.829 (0.801–0.858)	0.620 (0.549–0.695)	0.912 (0.885–0.936)	0.735 (0.663–0.801)	0.859 (0.827–0.887)	0.132 (0.113–0.150)
Random forest	0.841 (0.805–0.874)	0.831 (0.803–0.859)	0.564 (0.492–0.634)	0.936 (0.913–0.957)	0.777 (0.695–0.843)	0.845 (0.815–0.872)	0.129 (0.113–0.146)
AdaBoost	0.845 (0.809–0.880)	0.809 (0.779–0.838)	0.570 (0.500–0.642)	0.903 (0.874–0.929)	0.699 (0.617–0.777)	0.842 (0.811–0.874)	0.190 (0.186–0.195)
Logistic regression	0.810 (0.769–0.845)	0.810 (0.779–0.839)	0.492 (0.414–0.564)	0.936 (0.914–0.956)	0.752 (0.667–0.821)	0.824 (0.787–0.854)	0.142 (0.126–0.160)
Extra trees	0.811 (0.773–0.847)	0.806 (0.774–0.835)	0.603 (0.536–0.671)	0.885 (0.855–0.912)	0.675 (0.597–0.749)	0.850 (0.818–0.881)	0.158 (0.136–0.181)
Naive Bayes	0.808 (0.770–0.846)	0.701 (0.665–0.735)	0.754 (0.696–0.815)	0.681 (0.639–0.720)	0.482 (0.420–0.539)	0.875 (0.842–0.908)	0.230 (0.202–0.257)
Neural network	0.806 (0.765–0.845)	0.799 (0.769–0.828)	0.480 (0.418–0.555)	0.925 (0.902–0.946)	0.717 (0.638–0.789)	0.819 (0.788–0.850)	0.144 (0.127–0.161)
SVC	0.806 (0.765–0.846)	0.803 (0.773–0.837)	0.430 (0.359–0.509)	0.949 (0.931–0.969)	0.770 (0.691–0.854)	0.809 (0.774–0.845)	0.153 (0.133–0.170)
K‐nearest neighbours	0.803 (0.766–0.844)	0.799 (0.768–0.829)	0.587 (0.514–0.651)	0.883 (0.854–0.909)	0.665 (0.592–0.732)	0.844 (0.812–0.873)	0.154 (0.135–0.174)
Decision tree	0.765 (0.715–0.812)	0.814 (0.782–0.844)	0.564 (0.497–0.638)	0.912 (0.884–0.937)	0.716 (0.639–0.787)	0.841 (0.806–0.874)	0.157 (0.137–0.185)

*Note*: Data are presented as point estimate (95% confidence interval).

Abbreviations: AUC, area under the receiver operating characteristic curve; CI, confidence interval; NPV, negative predictive value; PPV, positive predictive value; SVC, support vector classifier.

CatBoost had the marginally highest cross‐validated AUC during training (0.861) and test‐set AUC (0.858; 95% CI 0.829–0.892). gradient boosting (AUC 0.857), XGBoost (0.857) and LightGBM (0.850) performed similarly, and the DeLong comparison between CatBoost and gradient boosting was not significant (*p* = 0.953). CatBoost was therefore selected as the index model for detailed description on the basis of a small numerical advantage; these results do not establish superiority over the other leading gradient‐boosting models.

### 3.3 Detailed performance of the selected CatBoost model

**FIGURE 3:**
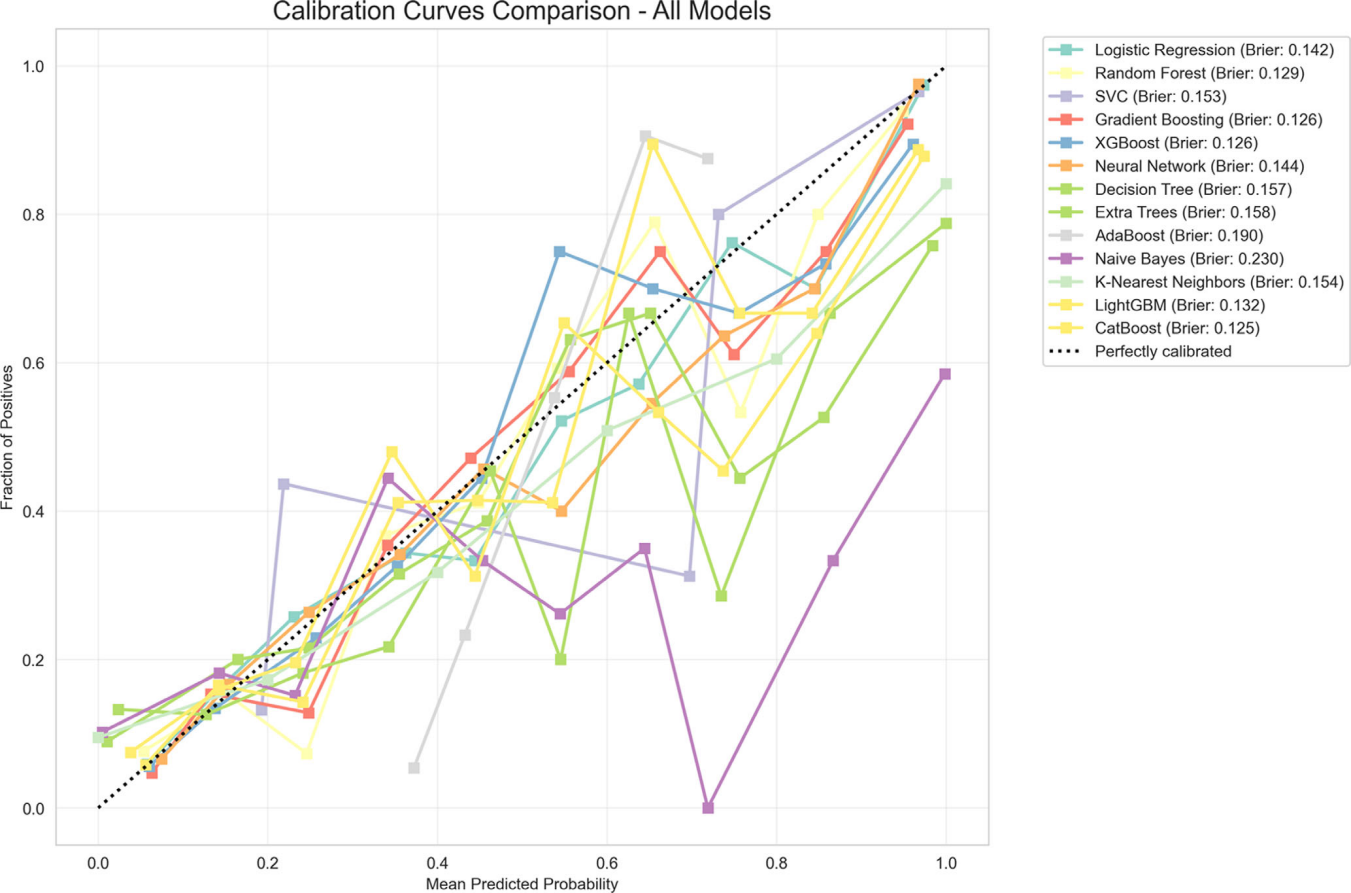
Calibration curves for all models.

The selected primary CatBoost model had sensitivity 0.587 (95% CI 0.513–0.662), specificity 0.930 (0.903–0.951), PPV 0.766 (0.693–0.833), NPV 0.851 (0.821–0.882), LR + 8.32, LR − 0.44 and Brier score 0.125 (Table 3). High specificity indicates relatively few positive predictions among culture‐negative records; it does not establish safe rule‐out, and specificity alone should not be interpreted as PPV. Given the moderate sensitivity and LR− of 0.44, a negative or low‐probability result reduces but does not exclude culture positivity. PPV and NPV also depend on the 28.3% outcome prevalence in this cohort. The Brier score measures overall probabilistic accuracy but does not by itself establish calibration. In the separately reconstructed CatBoost analysis (AUC 0.869; Brier score 0.117), mean predicted probability was 0.298 versus an observed proportion of 0.283; joint logistic recalibration yielded a slope of 0.765 and intercept of −0.325, while the offset‐only calibration‐in‐the‐large intercept was −0.151. The slope below 1 indicates that probabilities were too extreme, with modest overall overprediction. These calibration estimates pertain to the reconstructed model, not the archived primary model represented in Tables 2, 3, 4 and Figure 3.

**TABLE 3:** Confusion matrix and performance metrics for the selected primary CatBoost model (*n* = 633).

Parameter	Value (95% CI)
True negative (TN)	422
False positive (FP)	32
False negative (FN)	74
True positive (TP)	105
Sensitivity (recall)	0.587 (0.513–0.662)
Specificity	0.930 (0.903–0.951)
Positive predictive value (PPV)	0.766 (0.693–0.833)
Negative predictive value (NPV)	0.851 (0.821–0.882)
Positive likelihood ratio (LR^+^)	8.32 (6.01–12.19)
Negative likelihood ratio (LR^−^)	0.44 (0.37–0.52)
Diagnostic odds ratio (DOR)	18.71 (12.34–30.52)

*Note*: Test set size *n* = 633. Values in parentheses are 95% confidence intervals.

Abbreviations: DOR, diagnostic odds ratio; FP, false positive; FN, false negative; LR^−^, negative likelihood ratio; LR^+^, positive likelihood ratio; TN, true negative; TP, true positive.

**TABLE 4:** Exploratory distribution of culture positivity across primary model probability bands (*n* = 633).

Probability band	Probability range	Number of sample records (%)	Observed positive cultures (*n*)	Observed positive rate (%)
Lower predicted probability	< 0.20	379 (59.9%)	37	9.8%
Intermediate predicted probability	0.20–0.60	134 (21.2%)	44	32.8%
Higher predicted probability	> 0.60	120 (19.0%)	98	81.7%

*Note*: Probability bands summarise the archived primary CatBoost output and are exploratory, not validated treatment thresholds. The observed positive rate is the proportion of records in each band with a positive source culture label. No clinical action is recommended on the basis of these data. Percentages may not sum to 100% because of rounding.

### 3.4 Key predictors of urine‐culture positivity

**FIGURE 4:**
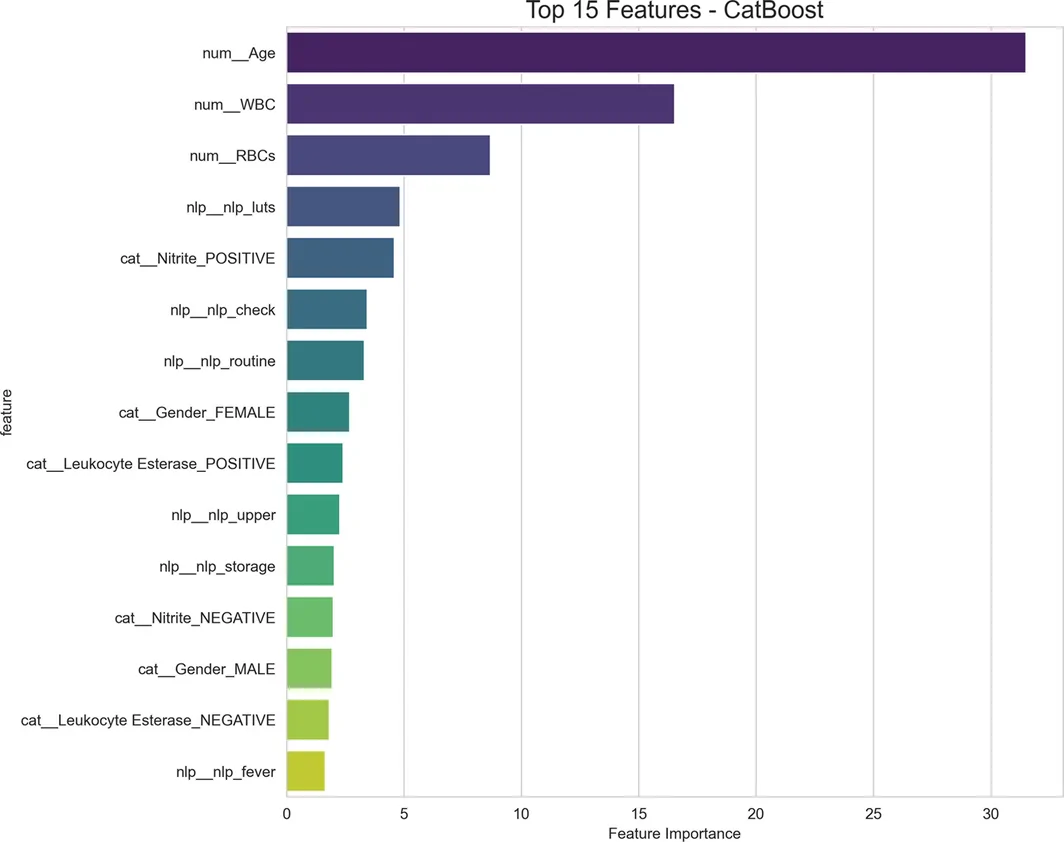
Top 15 most important features for the CatBoost model.

Feature‐importance analysis ranked age and several urinalysis variables, including leukocyte esterase, WBC, nitrite and RBC, among the influential predictors (Figure 4). Feature importance is descriptive and does not by itself demonstrate incremental predictive value, causality, biological validity or transportability.

In the post hoc reconstructed analysis, the full CatBoost model had AUC 0.869 (95% CI 0.832–0.899), compared with 0.848 (0.811–0.880) without text‐derived features (difference 0.021, 95% CI 0.000–0.041; paired‐bootstrap *p* = 0.050). Comparator AUCs were 0.770 (0.726–0.810) for a urinalysis‐only CatBoost model, 0.751 (0.707–0.791) for a dipstick‐only logistic model and 0.816 (0.777–0.854) for full multivariable logistic regression. These findings suggest a modest incremental contribution from the available text fields in this reconstruction; they are not estimates from the exact fitted primary model and require independent confirmation (Table S3).

Age distributions differed between outcome groups: median age was 13 years (IQR 2.5–44.5) in positive‐labelled records and 10 years (IQR 5–34) in negative‐labelled records (Figure S2). This association may reflect both biology and the age and test‐ordering composition of the cohort and should not be interpreted as evidence of causation.

### 3.5 Exploratory probability‐stratum and subgroup analyses

The archived primary CatBoost probabilities were summarised using the three exploratory probability bands. The analysis assessed statistical separation of recorded culture outcomes only; it did not evaluate decision‐curve net benefit, antibiotic use, patient‐important outcomes or the safety of acting on these categories.

**FIGURE 5:**
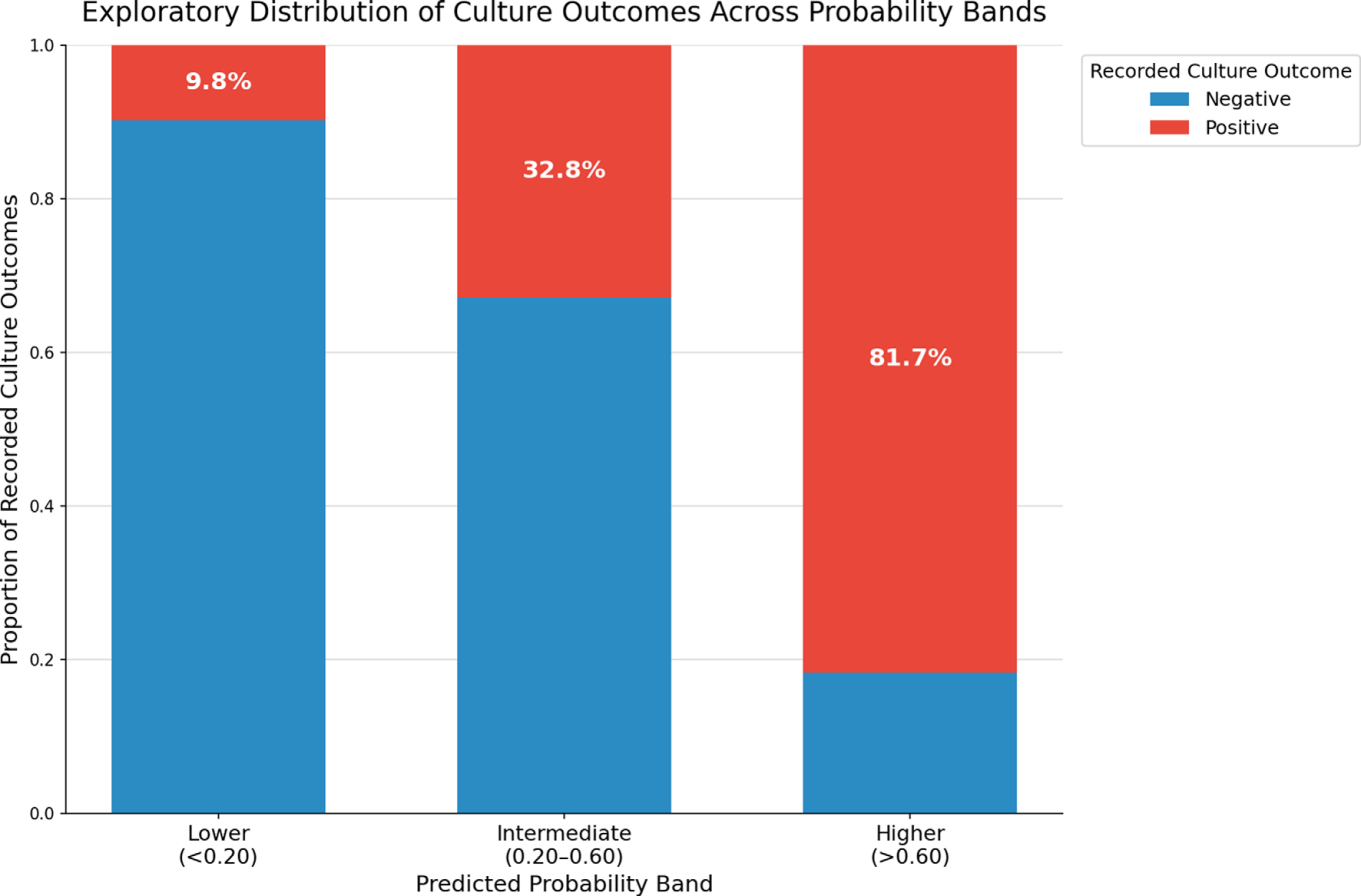
Exploratory distribution of recorded culture outcomes across primary model probability bands.

The lower probability band contained 379 of 633 test records (59.9%), including 37 positive cultures (9.8%). The intermediate band contained 134 records (21.2%), including 44 positive cultures (32.8%), and the higher probability band contained 120 records (19.0%), including 98 positive cultures (81.7%) (Table 4; Figure 5). These values demonstrate descriptive risk separation only. The primary individual prediction vector was not retained in the supplied archive, and the counts differ from those obtained with the separately reconstructed model; accordingly, no treatment, withholding or follow‐up recommendation is assigned to any probability band.

In the post hoc reconstructed analysis, age‐stratum performance at the fixed training‐derived threshold was <12 years, *n* = 328 (79 positive), AUC 0.896 (95% CI 0.846–0.937), sensitivity 0.684 and specificity 0.912; 12–17 years, *n* = 57 (11 positive), AUC 0.619 (0.454–0.779), sensitivity 0.091 and specificity 0.913; 18–64 years, *n* = 212 (67 positive), AUC 0.887 (0.835–0.935), sensitivity 0.746 and specificity 0.855; and ≥65 years, *n* = 36 (22 positive), AUC 0.756 (0.582–0.911), sensitivity 0.864 and specificity 0.429. The adolescent and older‐adult strata were small, all estimates were internally derived, and the results do not constitute subgroup validation.

## 4 DISCUSSION

### 4.1 Principal findings

This study compared 13 machine‐learning models for predicting the supplied urine‐culture label in a predominantly paediatric, test‐ordered cohort. Several gradient‐boosting models showed similar internal discrimination, with CatBoost selected as the index model because of a marginal numerical advantage. The model estimates culture positivity after urinalysis results and recorded text are available in patients who have already had a culture ordered. Its present role is therefore limited to diagnostic risk estimation; it was not evaluated as a rule for deciding who should undergo culture, diagnosing symptomatic UTI or selecting, starting, withholding or delaying antibiotics.

### 4.2 Clinical performance and interpretation

The primary CatBoost model had high specificity and LR+, supporting identification of records with an increased probability of culture positivity. Conversely, sensitivity was 0.587, and the exploratory lower probability band still contained 37 positive cultures. A low estimate therefore cannot rule‐out culture positivity, and a high estimate does not establish symptomatic UTI because positive cultures can reflect asymptomatic bacteriuria, colonisation or contamination. Treatment decisions require symptoms, illness severity, host factors and alternative diagnoses and were outside the scope of this study.

### 4.3 Benchmarking against existing literature

The primary CatBoost AUC of 0.858 falls within the range reported by prior studies, although cross‐study comparisons are indirect because populations, outcome definitions, predictors and validation designs differ. Taylor et al. reported an AUC of 0.904 in emergency‐department visits.^18^ Recent studies by Sergounioti et al. reached 0.819 on 8000 urinalysis records, while Demirci et al. achieved 0.822 on 12 000 patients.^19^, ^20^ A structured comparison is provided in Table S1.

Across published studies, predictive performance varies with the target population, outcome definition, predictor availability and validation design. The recurring importance of nitrite, leukocyte esterase, urinary WBC and related urinalysis variables provides face validity but does not establish a universal performance plateau or show that a model will transport to a different clinical setting.

This clinical intuition is supported by prior research. Sergounioti et al. similarly highlighted positive nitrites, white blood cell count and specific gravity as critical predictors.^19^ Demirci et al. used SHAP analysis and identified urinary leukocytes, nitrite, and bacterial count as top predictors.^20^ Agreement in influential urinalysis variables provides face validity, but it does not prove causality or transportability. Age, urinalysis and text features may also encode local case mix and test‐ordering behaviour.

### 4.4 Applicability and transportability

Prior studies have examined different clinical targets and populations: Taylor et al. modelled positive urine‐culture results in adult emergency‐department visits, Choi et al. evaluated urinalysis‐plus‐AI prediction of culture results in large laboratory cohorts, and Yen et al. predicted critical deterioration rather than culture positivity in adults with UTI.^18^, ^21^, ^22^ Xiong et al. developed a UTI‐risk model specifically in hospitalised adults with type 2 diabetes.^23^

The study population was a heterogeneous, predominantly paediatric test‐ordered cohort rather than a symptom‐defined UTI cohort. Broad inclusion may reflect local test‐ordering patterns, but it does not ensure applicability to adult urology, paediatric emergency care, inpatients, catheterized patients, pregnancy or other specific populations. Although records originated from three hospitals, they were pooled before a sample‐record random split; centre‐specific and temporal validation was impossible, and dependence among repeated file numbers was not controlled. Robustness, broad applicability and generalizability should not be inferred without patient‐grouped external validation.

### 4.5 Clinical context, guidelines and investigational role

Guideline‐concordant management depends on clinical syndrome, illness severity, host factors and specimen context—not predicted culture positivity alone. In suspected systemic UTI, complicated UTI, pyelonephritis or sepsis, urine should be obtained for culture and susceptibility testing when feasible and empirical antimicrobial therapy initiated without avoidable delay. Empirical selection should consider illness severity, resistance risk, previous cultures and antibiotic exposure, patient‐specific factors and local susceptibility data. NICE recommends immediate antibiotic treatment for symptomatic lower UTI in pregnant women and men and for children and young people younger than 16 years; urine should be collected before treatment when feasible, but treatment should not be delayed in a seriously ill patient.^5^, ^7^, ^8^, ^9^

Back‐up antibiotic prescribing is a narrower strategy. NICE recommends considering immediate or back‐up treatment only for nonpregnant women with lower UTI when immediate antibiotics are not judged necessary, taking account of symptom severity, complication risk, prior cultures and antibiotics and patient preference. A back‐up prescription is used if symptoms fail to begin improving within 48 h or worsen at any time. The supporting evidence was derived from nonpregnant women; NICE identified no randomised evidence for this strategy in men, pregnant women, older people or children. It should not be extrapolated to this predominantly paediatric cohort or to patients with systemic illness, pregnancy, a catheter, immunosuppression or structural or functional urinary‐tract abnormalities.^5^, ^24^

Asymptomatic bacteriuria is a separate no‐treatment scenario, not delayed treatment. In older or catheterized patients with bacteriuria but no attributable urinary or systemic symptoms, guidelines generally recommend against screening or antimicrobial treatment, with exceptions such as pregnancy and selected endoscopic urological procedures involving mucosal trauma. Conversely, compatible symptoms in a catheterized patient indicate possible catheter‐associated UTI and warrant clinical assessment, appropriate culture and treatment without waiting for the final result when antibiotics are indicated. A culture‐positivity model cannot identify asymptomatic bacteriuria because ASB requires bacteriuria plus absence of attributable symptoms, and symptom attribution and catheter status were unavailable here.^4^, ^6^, ^7^, ^10^

No subgroup in this retrospective study can be declared safe for antibiotic delay on the basis of model output. Published prediction studies have reported high NPVs, but these estimates are prevalence‐ and threshold‐dependent and do not establish treatment safety. Demirci et al. reported an NPV of 90.4%, while Sergounioti et al. achieved 96.4% NPV by optimising decision thresholds, potentially reducing overtreatment by 56%.^19^, ^20^ These comparisons do not demonstrate that antibiotic use can safely be reduced in the present cohort.

CatBoost has been reviewed across large‐data applications, but method‐level evidence does not establish clinical utility in this cohort.^25^ Separate studies have applied machine learning to antimicrobial‐resistance prediction and prescribing.^26^, ^27^, ^28^ These models require pathogen and susceptibility targets and are distinct from the present culture‐positivity model. The present study did not model susceptibility or prescribing and should not be described as a gatekeeper for such systems. A structured overview of selected machine‐learning applications in antimicrobial‐resistance prediction is provided in Table S2.

Available diagnostic strategies answer different questions. Dipstick urinalysis is rapid but does not identify an organism or provide susceptibility; laboratory culture provides organism identification and phenotypic susceptibility but usually requires 24–72 h. Automated flow cytometry, direct‐urine MALDI‐TOF, molecular assays, biosensors and point‐of‐care culture systems can shorten parts of this pathway, but performance, susceptibility capability, cost and implementation requirements vary. NICE evaluated 12 point‐of‐care technologies and did not recommend any for early routine NHS use, while evidence on accuracy and prescribing impact remains insufficient; culture‐based systems requiring approximately 16–24 h were considered too slow to improve primary‐care prescribing. The present model uses existing urinalysis and recorded text, but it neither identifies an organism nor provides susceptibility, does not shorten culture processing, and has unmeasured implementation cost. It is investigational and complementary to—not a replacement for—microbiological or point‐of‐care testing.^29^, ^30^

### 4.6 Strengths and limitations

Strengths include comparison of 13 algorithms, use of a multicentre‐source real‐world dataset, reporting of multiple discrimination measures with confidence intervals and evaluation of structured and text‐derived predictors. These strengths do not remove the limitations of sample‐record internal validation.

First, the cohort was selected by the joint occurrence of urinalysis and culture testing and did not represent all patients presenting with possible UTI. The source records included routine testing and non‐urinary complaints, but the dataset lacked the denominator of potentially eligible presentations, clinician‐stated reasons for testing, care setting, prescriptions and treatment timing. We therefore cannot determine how frequently testing was used, whether it was guideline‐concordant, or whether treatment was usually empirical or culture‐directed. Past medical history was incompletely populated free text—documented in 290 records (11.5%) and blank in 2240 (88.5%)—so reliable comorbidity prevalence and comorbidity‐specific performance could not be calculated. Results apply only to similarly test‐ordered records.

Second, the reference outcome was the supplied culture label rather than clinically adjudicated UTI. Laboratory thresholds, specimen type, contamination rules and organism‐, age‐, sex‐ and catheter‐specific criteria were unavailable; some positive‐labelled records had low colony counts, fungal isolates, multiple organisms or missing microbiological detail. Prior antibiotic exposure and its timing were also unavailable. The model cannot distinguish symptomatic infection from asymptomatic bacteriuria, colonisation or contamination.

Third, the unit of analysis was the sample record rather than a confirmed unique patient or encounter. Repeated file numbers occurred, and reconstruction of the stated split found 71 file numbers in both training and test partitions, affecting 85 of 633 test records. Because cross‐centre identity and encounter dates were unavailable, this does not prove that the same patients crossed partitions, but within‐person correlation and patient‐level leakage cannot be excluded. Centre, date and setting identifiers were absent, preventing centre‐stratified, leave‐one‐centre‐out, temporal and setting‐specific validation; performance estimates may therefore be optimistic.

Fourth, data‐quality and reproducibility limitations were identified. Raw predictors contained missing values, age was stored using mixed units, and one gender entry was malformed. The text fields were sparse, lacked timestamps and had no dedicated handling of negation, abbreviations, spelling variation or language. The supplied workbook contains the de‐identified record‐level source fields and outcome labels, but not the original split assignments, fitted preprocessing/vectoriser and model objects, hyperparameter‐search results, record‐level predictions, bootstrap resamples or original analysis code. Consequently, the archived primary AUCs, calibration estimates, probability‐band counts, ablation results and subgroup metrics cannot be independently reproduced from the workbook alone. Reviewer‐requested subgroup, ablation, baseline and calibration analyses therefore used a separately specified fixed‐parameter reconstruction that produced AUC 0.869 rather than the primary 0.858; these are secondary robustness analyses, not verification or extension of the exact fitted primary model.

Finally, the probability cut‐points were exploratory and their original provenance could not be recovered; neither decision‐curve analysis nor prospective impact testing was performed. The study recorded no antibiotic exposure, symptom progression, re‐attendance, pyelonephritis, sepsis, admission, delayed treatment or treatment failure. It therefore provides no direct evidence that starting, withholding or delaying antibiotics on the basis of model output is safe or beneficial in any patient group. These findings should be regarded as hypothesis‐generating until reproducible patient‐grouped external validation and prospective clinical‐impact evaluation are completed.

### 4.7 Future directions

Future studies should preserve stable patient, encounter, centre, collection‐date, note‐timestamp, specimen type, symptom, catheter, pregnancy, treatment, susceptibility and outcome variables; use patient‐grouped development and geographic or temporal external validation; and assess calibration and clinical net benefit using prospectively specified thresholds. Prospective impact studies should evaluate antibiotic exposure together with patient‐important safety outcomes and should predefine eligibility, clinical actions, escalation rules and follow‐up.

Separate antimicrobial‐resistance prediction would require pathogen and susceptibility labels and a distinct development and validation pathway. It is outside the scope of the present culture‐positivity model and should not be inferred from these results.

## 5 CONCLUSION

In this sample‐record internal validation, several gradient‐boosting models discriminated between positive and negative source culture labels, with no significant AUC difference among the leading models. The selected CatBoost model warrants further evaluation only as a diagnostic risk‐estimation tool after urinalysis is available in patients who have already had culture ordered. It does not diagnose clinical UTI, distinguish asymptomatic bacteriuria or contamination or establish the safety or benefit of antibiotic treatment or deferral. Patient‐grouped external validation, reproducible laboratory outcome definitions, subgroup calibration and prospective evaluation of prescribing and patient‐safety outcomes are required before clinical use.

## AUTHOR CONTRIBUTIONS

Authors' contributions: Isaac Samir Wasfy contributed to conceptualization, methodology, investigation, data curation, writing—original draft, and visualisation; Jamal Ahmad contributed to conceptualization, methodology, software, formal analysis, investigation, writing—original draft and visualisation; Hany Elsegeay, Mohamed F. Elebiary, Ahmed Haty, Eman M. El‐Dydamony, Ahmed Mohamed Soliman, Ahmed Alrefaey, Hesham Abozied, Mohamed Algammal, Hossam A. Shouman, Maha M. Elzamek, Ahmed Abdel Galil Saleh, Ahmed Fetyan Abdelazim Shafi and Osama Mostafa Abdalla Mohamed contributed to investigation, data curation and writing—review and editing. All authors contributed to the interpretation of the data, revised the manuscript critically for important intellectual content and approved the final version for submission.

## CONFLICT OF INTEREST STATEMENT

The authors declare no conflicts of interest.

## Supporting information


**Figure S1** Heat map of Performance Metrics Across All Models.

**Figure S2** Violin Plot of Patient Age Distribution by Urine Culture Outcome.

**Table S1** Selected machine‐learning studies in UTI‐ and urine‐culture‐related cohorts.

**Table S2** Selected clinical applications of machine learning for antimicrobial‐resistance prediction and empirical antibiotic selection.

**Table S3** Post hoc reconstructed feature‐ablation and simpler model comparisons (test‐set AUC).

## Data Availability

The de‐identified source workbook and Tables S1–S3 are supplied for editorial and peer‐review assessment. The workbook permits verification of cohort totals, age groups, repeated identifiers, missingness and microbiological summaries, but it is not a complete model‐reproducibility package and does not contain the original split manifest, fitted preprocessing/model objects, predictions, resamples or analysis code. Additional de‐identified data may be available from the corresponding author on reasonable request and subject to approval by the Al‐Azhar University Hospitals Institutional Review Board.
