# Supplementary material for: Machine‐learning prediction of urine‐culture positivity in a multicentre test‐ordered cohort: Model development and internal validation
Source: BJUI Compass. 2026 Sep 8;7(9):e70280. doi: 10.1002/bco2.70280 (PMC13552025; doi:10.1002/bco2.70280)
Supplement: Supplementary file 1 [file BCO2-7-e70280-s001.docx]

**Supplementary Table 3. Post hoc reconstructed feature-ablation and simpler-model comparisons (test-set AUC).**

| **Model (features)** | **AUC (95% CI)** | **Comment** |
| --- | --- | --- |
| CatBoost - full (age/sex + urinalysis + NLP) | 0.869 (0.832-0.899) | Post hoc reconstructed full model |
| CatBoost - without NLP (age/sex + urinalysis) | 0.848 (0.811-0.880) | Delta AUC vs full = 0.021 (95% CI 0.000-0.041; paired-bootstrap p=0.050) |
| CatBoost - urinalysis only (dipstick + WBC + RBC) | 0.770 (0.726-0.810) | No demographics or text-derived features |
| Logistic regression - full feature set | 0.816 (0.777-0.854) | Simple linear benchmark |
| Logistic regression - dipstick only (blood/nitrite/LE) | 0.751 (0.707-0.791) | Minimal dipstick benchmark |

Note: These analyses were conducted post hoc in response to peer review using a separately specified fixed-parameter reconstruction on the study dataset (n=2,530; 715 positive). A reconstructed 75%/25% stratified sample-record split was used; it is not an archived copy of the original primary split. Unit-aware age parsing was applied, and imputation, scaling, categorical transformations, and the TF-IDF vocabulary were fitted on the training partition only and then applied unchanged to the hold-out test set (n=633). AUC confidence intervals used 2,000 bootstrap resamples; the paired full-versus-without-NLP comparison used 3,000 resamples. The reconstructed full-model AUC (0.869) differs from the archived primary result (0.858), so these values are secondary reconstruction results and cannot verify the exact fitted primary model. Abbreviations: AUC, area under the receiver operating characteristic curve; CI, confidence interval; NLP, natural language processing; WBC, white blood cell; RBC, red blood cell; LE, leukocyte esterase.
