# Supplementary material for: Machine‐learning prediction of urine‐culture positivity in a multicentre test‐ordered cohort: Model development and internal validation
Source: BJUI Compass. 2026 Sep 8;7(9):e70280. doi: 10.1002/bco2.70280 (PMC13552025; doi:10.1002/bco2.70280)
Supplement: Supplementary file 2 [file BCO2-7-e70280-s002.docx]

**Supplementary Table 1. Selected machine-learning studies in UTI- and urine-culture-related cohorts.**

| **Study** | **Population/setting and prediction target** | **Key predictors** | **Leading method** | **Reported discrimination** |
| --- | --- | --- | --- | --- |
| Taylor et al. (2018) [ref 18] | 80,387 adult emergency-department visits with urine-culture results and UTI-attributable symptoms; target: positive urine-culture/UTI label | Demographics, symptoms, vital signs, laboratory data, medications, medical history, and structured examination findings | XGBoost (full model) | AUC 0.904 (95% CI 0.898-0.910) |
| Xiong et al. (2024) [ref 23] | 1,340 hospitalized adults with type 2 diabetes (440 UTI cases and 900 controls); target: UTI label | Demographic, comorbidity, medication, and clinical/laboratory variables | Gradient boosting | AUC 0.9789 |
| Yen et al. (2024) [ref 22] | 3,622 emergency-department visits among 3,235 adults with UTI; target: in-hospital death or ICU transfer within 12 hours | Twenty selected clinical/laboratory variables | XGBoost | AUC 0.833 |
| Sergounioti et al. (2025) [ref 19] | 8,065 hospital-laboratory urine-sample records; target: culture positivity (>10^5 CFU/mL in that study) | Demographic and routine urinalysis variables | XGBoost | AUROC 0.819 (abstract value) |
| Demirci et al. (2025) [ref 20] | 12,433 patients who underwent urinalysis, urine culture, complete blood count, and CRP testing; target: culture positivity | Urinalysis and hemogram data | Gradient boosting (H2O AutoML) | AUC-ROC 0.822 |
| Present study | 2,530 urine-sample records from three university hospitals; target: supplied culture label | Age/sex, urinalysis, and text-derived features | CatBoost index model | AUC 0.858 (95% CI 0.829-0.892); sample-record internal validation |

Note: Targets, cohorts, predictors, and validation designs differ; values are not directly comparable. References correspond to the manuscript reference list.

For Sergounioti et al., the article reports non-identical AUROC values across the abstract and displayed tables; the abstract value used in the manuscript is shown here.
