# Supplementary material for: Machine‐learning prediction of urine‐culture positivity in a multicentre test‐ordered cohort: Model development and internal validation
Source: BJUI Compass. 2026 Sep 8;7(9):e70280. doi: 10.1002/bco2.70280 (PMC13552025; doi:10.1002/bco2.70280)
Supplement: Supplementary file 3 [file BCO2-7-e70280-s005.docx]

**Supplementary Table 2. Selected clinical applications of machine learning for antimicrobial-resistance prediction and empirical antibiotic selection.**

| **Study** | **Setting/population** | **Prediction target** | **Method** | **Main reported finding** |
| --- | --- | --- | --- | --- |
| Yelin et al. (2019) [ref 26] | Ten-year Israeli health-system dataset of >700,000 community-acquired UTI records linked to >5,000,000 antibiotic-purchase records | Patient-specific resistance to six antibiotics | Logistic regression and gradient-boosted decision trees using demographics, prior cultures, and antibiotic-purchase history | Retrospective personalized recommendations reduced mismatched empirical treatment compared with observed standard care. |
| Corbin et al. (2022) [ref 27] | 8,342 infections from Stanford emergency departments and 15,806 uncomplicated UTI episodes from two Boston hospitals | Antibiotic susceptibility/activity of candidate empirical regimens | Personalized antibiograms (logistic regression, random forest, and LightGBM) plus linear-programming selection | Coverage was 85.9% vs 84.3% for clinicians at Stanford (p=0.11) and 90.4% vs 88.1% in Boston (p<0.0001); at clinician-level coverage, 48% of ciprofloxacin selections in Boston could be shifted to trimethoprim/sulfamethoxazole in simulation. |
| Yuan et al. (2025) [ref 28] | 4,709 Enterobacterales bloodstream-infection episodes in Oxfordshire, UK; training in 2017-2021 and temporal testing in 2022-2023 | Resistance to seven antibiotics | XGBoost | Test AUCs were 0.680-0.737 without species identification and 0.723-0.827 with species; retrospective simulations yielded active beta-lactam treatment for 79% vs 70% under clinician prescribing at similar overtreatment. |

Note: These studies predict resistance or susceptibility after an infection/organism context is available and are distinct from the present culture-positivity model. Findings are retrospective and do not establish prospective clinical benefit. References correspond to the manuscript reference list.
